# A new anesthetic protocol to medullary nerve roots access in rats

**DOI:** 10.1590/ACB360908

**Published:** 2021-11-08

**Authors:** Deivid Ramos dos Santos, Renan Kleber Costa Teixeira, Nayara Pontes de Araújo, Faustino Chaves Calvo, Tiago Braga Duarte, Letícia Amanda Pinheiro de Ataíde, Rosa Helena de Figueiredo Chaves, Rui Sergio Monteiro de Barros

**Affiliations:** 1 Fellow Master degree. Postgraduate Program in Surgery and Experimental Research - Universidade do Estado do Pará (UEPA) – Belem (PA), Brazil.; 2 MD. Department of Experimental Surgery - School of Medicine, Universidade do Estado do Pará (UEPA) – Belem (PA), Brazil.; 3 Graduate student. School of Medicine - Universidade Federal do Pará (UFPA) – Belem (PA), Brazil. Conception, interpretation of data, manuscript writing; 4 Graduate student. School of Medicine - Universidade Federal do Pará (UFPA), Belem (PA), Brazil.; 5 Graduate student. School of Medicine - Universidade Federal do Pará (UFPA) – Belem (PA), Brazil.; 6 Graduate student. School of Medicine – Universidade do Estado do Pará (UEPA) – Belem (PA), Brazil.; 7 Fellow PhD degree. Postgraduate Program in Health and Animal Production in Amazon - Universidade Federal Rural da Amazônia (UFRA) – Belem (PA), Brazil.; 8 PhD, Associate Professor. Department of Experimental Surgery - School of Medicine - Universidade do Estado do Pará (UEPA) - Belem (PA), Brazil.

**Keywords:** Microsurgery, Anesthesia, Animal Experimentation, Rats

## Abstract

**Purpose:**

To describe a new anesthetic protocol medullary and nerve roots access and in *Rattus norvegicus*.

**Methods:**

Seventy female Wistar rats (n=70) were used. The animals were randomly divided into two laminectomy groups: cervical (n=40) and thoracic (n=30). In cervical group, a right posterior hemilaminectomy was performed to access the nerve roots. In thoracic group, a laminectomy of the eighth thoracic vertebra was accomplished. Thirty-five rats (20 cervical and 15 thoracic) were submitted to old anesthetic protocol (ketamine 70 mg/kg plus xylazine 10 mg/kg); and the 35 other animals (20 cervical and 15 thoracic) were submitted to a new anesthetic protocol (ketamine 60 mg/kg,xylazine 8 mg/kg and fentanyl 0.03 mg/kg).

**Results:**

The time to complete induction was 4.15 ±1.20 minin ketamine, xylazine and fentanyl group, and it was 4.09 ±1.47 min in the ketamine and xylazine group. There was no correlation in the time required to perform the cervical laminectomy in the old anesthetic protocol. In all groups, the animals submitted to the old anesthetic protocol had a higher level of pain on the first and third postoperative days than the animals submitted to the new anesthetic protocol.

**Conclusions:**

The new anesthetic protocol reduces the surgical time, allows better maintenance of the anesthetic plan, and brings more satisfactory postoperative recovery.

## Introduction

Spinal nerve avulsion injuries are devastating both from the functional and the psychosocial points of view, due to poor neural regeneration, especially the preganglionic lesions, that, as a rule, have the worst prognosis owing to their considerable failure rate^1^^-^ 3. They occur when the nerves that connect the spinal cord to the muscles are arranged (avulsed). As root avulsion is a longitudinal injury to the spinal cord, it differs from other peripheral nerve injuries and is beyond surgical repair^3^^,^ 4.

Avulsion injuries has grown as the number of motorcycle accidents increases^1^^,^ 2. Unfortunately, in 70% of these accidents, at least one root is completely avulsed, due to the high impact related to the biomechanics of trauma^2^^,^ 3.

Actually, the surgical strategy after root avulsion injury is palliative and culminates in deficient functional recovery. In the last 20 years, in only some experimental models, it has been reported that avulsioned root nerve neurotization is possible by reimplantation of the injured root in the spinal cord^5^^,^ 6.

Years later, the first clinical cases submitted to replantation surgery for single roots in humans were described, and limited functional recovery of the shoulder and elbow was report^7^. The use of experimental avulsion models is one of the key steps to obtain good functional results in this repair^7^^-^ 10.

Although laminectomy is a widely performed technique, surgical description and an anesthetic protocol that brings better postoperative recovery are, in most cases, neglected and rarely reported.

Therefore, this research can contribute to the improvement of experimental surgical techniques for implantation of avulsed roots and their subsequent studies to assess neural regeneration. In this work, a new anesthetic protocol medullary and nerve roots access and in *Rattus norvegicus* was described.

## Methods

This research followed the rules of the Brazilian Law for Animal Care (Law no. 11.794/08) and Animal Research: Reporting of In Vivo Experiments (ARRIVE) guideline. It was approved by the Animal Use and Care Committee at Universidade do Estado do Pará (28/18).

Seventy female Wistar rats (n=70) obtained from the Animal Colony of Instituto Evandro Chagas were used. The sample size was decided intentionally by the researchers. The animals were kept in a vivarium of the Experimental Surgery Laboratory at Universidade do Estado do Pará (Brazil) with a controlled environment. Water and food were provided *ad libitum*.

The animals were randomly divided into two groups: cervical (n = 40) and thoracic (n = 30). In cervical group (CLG), a right posterior laminectomy surgery was performed, to access the nerve roots and simulate a brachial plexus avulsion model of to C7 injury. In thoracic group (TLG), the animals were submitted to a partial spinal cord injury.

The procedures were performed under video system^11^^,^ 12, composed by a high-definition Sony^©^ camcorder DCR-SR42 set to 52× magnification connected with a 4K 55-inch curved television by a digital HDMI cable. Two-low intensity halogen light source were used near to the camera to provide adequate illumination of the operative field.

Thirty-five rats (20 cervical and 15 thoracic) were submitted to old anesthetic protocol (OAP)^13^^-^ 15, with ketamine (K) 70 mg/kg plus xylazine (X) 10 mg/kg, intraperitoneal, proving to be effective during the operative period without intercurrences, as used in the experimental surgery laboratory^16^^,^ 17. The other 35 animals (20 cervical and 15 thoracic) were submitted to a new anesthetic protocol (NAP), with ketamine 60 mg/kg, xylazine 8 mg/kg and fentanyl 0.03 mg/kg, intraperitoneal^16^. Fentanyl was repeated for every 30 minutes until the end of the surgery.

To confirm the anesthesia, the withdraw reflex of the forelimb and hind limb and the reflexes of the eyelid and tail were observed using forceps. Surgical anesthetic depth was defined when the animals lost at least three out of the four reflexes^18^.

Confirmed the anesthesia, depending on the group, the cervical or thoracic region was shaved. Antisepsis was performed with povidone-iodine. The animals were placed in a horizontal ventral position above a plastic support on cervical or thoracic region to flex the spine to increase the interlaminar space.

A 4-cm incision was performed in the cervical or thoracic region above the column. The subcutaneous tissue was carefully laid back, and there was the blunt dissection of the paraspinal muscles to expose the cervical or thoracic spine. Meticulous hemostasis using bipolar cauterization were performed. Two-mL of lidocaine 2% was dripped in this area for better anesthetic management. The paraspinal muscles were pushed laterally with retractors. The spinous process of T2 was used as an anatomical landmark to identify the other vertebras.

In both groups, the procedure ended with the suture of muscles with 5-0 nylon and skin using 4-0 nylon. The animals were followed up by 14 days postoperatively. In all groups, the animals received tramadol hydrochloride^13^ 4 mg/kg at 12/12 h for up to five days by subcutaneous, and lidocaine topical was used in the incision at 12/12 h also for up to five days. In all groups, enrofloxacin was administrated by subcutaneous at 10 mg/kg once a day until seven days, to prevent meningeal infection. It was not necessary to perform the bladder massage. The animals were housed in isolated cages after the procedure to avoid injuries and pressure ulcers.

The parameters analyzed were time to surgical access and Rat Grimace Scale^14^. The scale was performed at the first, third, seventh and fourth quarter postoperatively day. The software BioEstat^©^ 5.4 was used. All data were expressed as means ± standard deviation. The Student’s *t*-test was used to compare tests score by groups, and the Fisher’s exact test was applied to compare tests score by weight. Statistical significance was assumed at p< 0.05.

## Results

All animals survived during the study period.

In all groups, the time to complete induction was 4.15 ±1.20 min in the NAP group (ketamine, xylazine and fentanyl), and it was 4.09 ±1.47 min in the OAP group (ketamine and xylazine). There was not statistically difference (p = 0.73).

In CLG and TLG groups, the old protocol showed more signs of pain during the surgery (18/20 and 12/15, respectively) than the new protocol (3/20 and 3/15, respectively) (p<0.0001). The necessity of new anesthetic dose during the surgery was 10/40 in CLG (new anesthetic protocol: 3/20 *vs*. old protocol: 7/20; p<0.0001) and it was 8/30 in TLG (new anesthetic protocol: 2/20 *vs*. old protocol: 6/20; p<0.0001).

In CLG, the time required to complete each procedure was 41.35 ±5.67 min. When comparing it considering the anesthetic protocol, the time required was 38.16 ±5.95 min in the new protocol and it was 59.92 ±11.36 min in the old protocol. The difference between the groups was statistically significant (p = 0.03).

In TLG, the time required to complete each procedure was 41.35 ±5.67 min. When comparing it for anesthetic protocol, the time required was 38.16 ±5.95 min in the new protocol and it was 59.92 ±11.36 min in the old protocol. The difference between the groups was statistically significant (p = 0.02).

The correlation analysis between time and the order of surgeries showed reduction in the time required to perform the surgery (Pearson’s correlation coefficient = -0.72, 95% confidence interval – 95%IC = -0.39 – -0.85, p < 0.01) in the new anesthetic protocol. There was no correlation in the time required to perform the cervical laminectomy in the old anesthetic protocol.

When comparing the groups according to the Rat Grimace Scale^18^, the animals submitted to the old anesthetic protocol had a higher level of pain on the first and third postoperative days than the animals submitted to the new anesthetic protocol (p <0.0001). There were no significant differences between the groups on the 14th postoperative day (p> 0.05) (Table 1).

**Table 1:** Mean scores of Rat Grimace Scale according to the groups submitted to anesthetic protocol.

Parameter	Postoperatively day			
	First	Third	Seventh	Fourth quarter
ketamine 70 mg/kg plus xylazine 10 mg/kg				
Thoracic group	0.78 ±0.33	0.54 ±0.21	0.26 ±0.14	0.16 ±0.12
Cervical group	0.65±0.31	0.49 ±0.28	0.23 ±0.12	0.19 ±0.13
ketamine 60 mg/kg, xylazine 8 mg/kg and fentanyl 0.03 mg/kg				
Thoracic group	0.40±0.25	0.24 ±0.18	0.18 ±0.13	0.14±0.10
Cervical group	0.35±0.29	0.21 ±0.15	0.17 ±0.12	0.13±0.11

## Discussion

Experimental models of spinal cord access are clearly invasive and trigger perioperative pain. However, the transoperatory anesthetic protocol and appropriate postoperative analgesics are not performed or registered with rigor, although their correct application reduces the variation in results due to pain-induced stress^18^^-^ 21.

It is known that the adequate anesthetic protocol directly influences the surgical success and the recovery of the experimental model^8^. When incorrect, it can bring perioperative risks and strongly alter the results studied^19^, mainly in spine surgery, because of its considerable failure rate, neurological deficits in the postoperative period and pain^13^.

Although laboratory rats are one of the most used models in several research areas, there is still no unanimous anesthetic protocol for certain complex microsurgical procedures, such as posterior intradural access to the spinal cord^13^^,^ 20 ^-^ 23.

Ketamin is classified as a dissociative anesthetic agent and does not provide deep anesthesia when used by itself. For this reason, ketamin needs a combination of sedatives such as xylazine. The dose most used in rats is 80-100 mg/kg for ketamin and 10 mg/kg for xylazine. Fentanyl is a µ-opioid receptor agonist and, despite it was used to modulate intraoperative pain, its effect on pain modulation in spinal cord models has not yet been elucidated^24^^,^ 25.

In relation to the induction time, there was no statistical difference until complete induction between groups (P = 0.73). Although numerically the rats submitted to the new anesthetic protocol took a little longer, the animals received a lower dose of anesthetic, increasing operative safety.

The animals submitted to the new anesthetic protocol had less signs of pain during the operative procedure. On the other hand, those ones submitted to the combination of ketamine and xylazine needed new doses of anesthetics, increasing the risk of adverse events, such as hypothermia, bradycardia and respiratory depression^24^^,^ 25. In this sense, the new anesthetic protocol proves to be safer and allows a surgical procedure with reduced potential risks.

There was a decrease in operative time in all animals submitted to the new anesthetic protocol. This is mainly due to safer anesthesia, with an induction plan and lasting maintenance for the chosen surgical procedure.

The general operative time was longer in animals submitted to the old protocol. This is justified by the early superficialization of the animal, the need of new anesthetic administration, checking the absence of reflexes and, finally, restarting the surgery.

According to scores of Rat Grimace Scale, better postoperative recovery was found in animals submitted to the new anesthetic protocol, mainly in first and third days after surgery, similar to that reported in other studies^13^^,^ 25. The pain scale has high sensitivity in the immediate postoperative period and, although its decrease does not necessarily mean spontaneous pain resolution, its application serves to refine our understanding of the best surgical recovery of the experiments^24^^,^ 27.

## Conclusions

The new anesthetic protocol reduces the surgical time, allows better maintenance of the anesthetic plan, and brings more satisfactory postoperative recovery. Medullary nerve roots access needs to be performed by experienced researchers to avoid misinterpretation. Further studies are suggested using different anesthetic concentrations for a better understanding of the new protocol.
